# Overexpression miR-486-3p Promoted by Allicin Enhances Temozolomide Sensitivity in Glioblastoma Via Targeting MGMT

**DOI:** 10.1007/s12017-020-08592-5

**Published:** 2020-02-21

**Authors:** Henggang Wu, Xu Li, Tiehui Zhang, Guojun Zhang, Jingnan Chen, Li Chen, Min He, Bilie Hao, Cheng Wang

**Affiliations:** 1 Department of Neurosurgery, The Second Affiliated Hospital of Zhejiang Chinese Medical University, Hangzhou, 310011 Zhejiang China; 2 Department of Neurosurgery, Wenrong Hospital of Hengdian, Jinhua, 322118 Zhejiang China; 3 Department of Neurosurgery, The First Affiliated Hospital of Zhejiang Chinese Medical University, Hangzhou, 310002 Zhejiang China

**Keywords:** Allicin, O6-methylguanine-DNA methyltransferase (MGMT), Temozolomide (TMZ), Glioblastoma, miR-486-3p

## Abstract

Glioblastoma is the most common primary tumor of the central nervous system that develops chemotherapy resistance. Previous studies showed that Allicin could inhibit multiple cancer cells including glioblastoma, but the function of Allicin in glioblastoma is still unclear. Our work aimed to investigate the underlying molecular mechanism. The results showed that miR-486-3p levels were greatly increased in glioblastoma during Allicin treatment. Overexpression of miR-486-3p increased chemosensitivity to temozolomide (TMZ) in vitro and in vivo. O6-methylguanine-DNA methyltransferase (MGMT) was identified as a direct target of miR-486-3p, and miR-486-3p overexpression prevented the protein translation of MGMT. Moreover, overexpression of MGMT restored miR-486-3p-induced chemosensitivity to TMZ. Taken together, our studies revealed that Allicin could upregulate miR-486-3p and enhance TMZ sensitivity in glioblastoma. The results suggested that in the future, Allicin can be used as an adjuvant therapy with TMZ to improve the prognosis of patients, and miR-486-3p may be a potential target for glioblastoma treatment to improve the curative effects.

**Electronic supplementary material:**

The online version of this article (10.1007/s12017-020-08592-5) contains supplementary material, which is available to authorized users.

## Introduction

Glioblastoma (GBM) are the most common central nervous system tumors in the clinic, accounting for 46.6% of all primary central nervous system tumors (Ostrom et al. 2016; Bush et al. 2017). At present, the treatment to glioblastoma is mainly based on surgical resection, post-operative radiotherapy, and chemotherapy as adjuvant therapy. Adjuvant therapy can significantly prolong the median survival of glioblastoma patients and improve their quality of life.

Temozolomide (TMZ) has been the standard chemotherapy for newly diagnosed GBM for many years. TMZ has certain advantages over many existing alkylating agents because of its small molecular weight. Many studies have shown that the levels of drug in the brain and cerebrospinal fluid are approximately 30% to 40% of the plasma concentration. Although TMZ can prolong the survival time by a small margin, a considerable number of glioblastoma patients are insensitive to TMZ treatment or gradually develop secondary resistance. Therefore, glioblastoma resistance to TMZ is considered to be the underlying cause of chemotherapy failure and glioblastoma recurrence (Kang et al. 2004). The underlying mechanisms require investigation and solutions to the problem of drug resistance are being sought.

MicroRNAs (miRNAs) are involved in a variety of human diseases, which can regulate gene expression post-transcriptionally by binding to mRNA 3′-untranslated region (Iorio and Croce 2009; Omura et al. 2008). Previous study demonstrated that miR-486 is often downregulated in malignancies and considered to be a tumor-suppressor miRNA (Chou et al. 2019; Yang et al. 2019). In addition, miR-486 could be an effective biomarker in cancer diagnosis and prognosis (Jiang et al. 2018). According to Genetics Network Analysis, Plaisier et al. found that high expression of miR-486-3p may enhance the sensitivity of neuronal tumors to chemotherapeutic drugs (Plaisier et al. 2016). However, this hypothesis has not been proved, and the effects of miR-486-3p on chemotherapeutic sensitivity of glioblastoma to TMZ and the underlying mechanisms are unknown.

Allicin is an ubiquitously found ingredient in garlic and widely used as food supplements all over the world(Lawson and Wang 2005). Allicin is reported to induce cell death and inhibit proliferation in cancer cells (Borlinghaus et al. 2014). These studies also suggested that Allicin induced p53-mediated autophagy in human liver cancer cells (Chu et al. 2012). Although studies have found that Allicin can inhibit glioblastoma proliferation (Pan et al. 2018), whether Allicin can enhance the sensitivity of glioblastoma to TMZ is unknown. Therefore, this study selected glioblastoma cell lines U251-TR, which is resistant to TMZ, as a research object to explore whether Allicin can reverse the TMZ resistance and was also performed to investigate the mechanism.

## Materials and Methods

### Cell Lines and Cell Culture

The glioblastoma cell lines U251-MG and A172 were obtained from the Cell Bank of the Chinese Academy of Sciences (Shanghai, China). The cell line was cultured in Dulbecco's modified Eagle's medium (DMEM, Gibco, Carlsbad, CA, USA) containing 2 mM glutamine, 10% fetal calf serum (FBS), 100 U/ml penicillin and 100 µg/ml streptomycin (Sigma, St. Louis, MO, USA). Cells were maintained at 37˚C in a 5% CO_2_ incubator.

### Isolation and Culture of Primary Glioblastoma Cells

Fresh brain tumor samples were collected from adult patients at the time of surgical excision who had provided informed written consent, and the tumors were removed surgically according to guidelines approved by the Institutional Review Board of The Second Affiliated Hospital of Zhejiang Chinese Medical University. GBM cells were isolated as described in previous study (Shankar et al. 2014; Rahman et al. 2015). Tumor specimens were washed thoroughly with cold PBS and cut into 1mm^3^ pieces, then digested with trypsin (0.5%, V/M) for 20–40 min at 37 °C in a shaking water bath. Primary glioblastoma cells were gently resuspended in DMEM/F12 medium (2 mmol/l l-glutamine) supplemented with 0.05% BSA, 20 ng/ml bFGF, 10 ng/ml EGF, 50 U ml of penicillin, and 50 μg ml of streptomycin. The culture medium was refreshed every 2–3 days.

### Generation of Temozolomide-Resistant Glioblastoma Cell Lines

The parental U251-MG cells were exposed to 400 µM TMZ for 3 weeks to generate TMZ-resistant colonies (Xu et al. 2017). Initially, we cultured the cell line in 6-well plates and allowed them to adhere during overnight incubation at 37 °C. TMZ treatment was repeated every 24 h for 5 consecutive days, and the cells were then exposed to fresh TMZ every 3 days for a total of 3 weeks. The majority of the cells died, but a small population survived and propagated. The surviving colonies were selected and established as TMZ-resistant U251 (U251-TR) cell lines.

### RNA Extraction, Reverse Transcription, and Quantitative-PCR (q-PCR)

Total RNAs were isolated using TRIzol (Ambion) based on the manufacturer’s protocol. For mRNA analysis, the cDNA was synthesized using random hexamer primers and SuperScript III reverse transcriptase (Invitrogen, Carlsbad, CA). For miRNA analysis, the cDNA was synthesized using specific stem-loop RT primers and TaqMan MicroRNA Reverse Transcription Kit (Applied Biosystems). q-PCR analysis was used to detect the MGMT using Maestro Green Eva Green q-PCR Master Mix (Maestrogen) and the expression level of miR-486-3p using QuantiTect SYBR Green PCR System (QIAGEN), according to the manufacturer’s instructions on the ABI Step OnePlus Real-time PCR system (Applied Biosystems). GAPDH was used as the internal control (Table 1). All reactions were run in triplicate and relative expression levels were calculated as $2^{{ - \Delta \Delta C_{{\text{T}}} }}$ after normalization with internal control.

**Table 1:** Sequences of primers used for experiments in this study

Primer name	Sequence (5′–3′)
GAPDH	F: GAAGGTGAAGGTCGGAGT
	R: GAAGATGGTGATGGGATTTC
MGMT	F: ACCGTTTGCGACTTGGTACTT
	R: GGAGCTTTATTTCGTGCAGACC
miR-486-3p	F: GCGGGGCAGCTCAGTA
	R: CGGGGCAGCTCAGTACAGGAT

### Plasmid Construction and Luciferase Reporter Assay

The target genes of miR-486-3p were selected based on the TargetScan (https://www.targetscan.org/, Release 7.2: March 2018). MGMT was selected as the possible target gene of miR-486-3p. The entire 3′-UTR of MGMT fragment, containing target sequences of miR-486-3p, were PCR amplified and cloned into the pmirGLO firefly luciferase-expressing vector (Promega, WI, USA) according to the manufacturer’s instructions. The miR-486-3p binding site mutation vectors were also constructed by using Site-Directed Mutagenesis Kit (Stratagene, La Jolla, CA), and all the constructs were verified by DNA sequencing. For luciferase reporter assay, luciferase reporter vectors together with miRNA-mimics (PM-486-3p) and negative control (NC) were transfected into U251-TR cells using Lipofectamine RNAi MAX Transfection Reagent (Thermo Fisher). After 48 h, luciferase activities were detected using the Dual Luciferase Reporter Assay System (Promega) on the Orion L luminometer (Berthold), according to the manufacturer’s protocol. Renilla luciferase served as the control reporter for normalization.

### Cytotoxicity Assay

Cell viability was measured using a cell counting kit-8 colorimetric assay (CCK-8; Dojindo Laboratories, Kumamoto, Japan) according to the manufacturer’s instructions. The transfected or wild-type cells were seeded into 96-well plates (3000 cells/well) and incubated at 37 °C. After 24 h, 10 μL of CCK-8 reagent was added per well and the cells were maintained for 2 h. The optical density values were detected at 450 nm using a Multiskan Sky Microplate Spectrophotometer (Thermo Fisher Scientific). IC_50_ values were determined using GraphPad Prism software version 5.0 (GraphPad Software, San Diego, CA, USA).

### Cell Apoptosis Assay

For cell apoptosis assay, 2 × 10^5^ cells were seeded in 6-well plates and transfected with miR-486-3p mimics (PM-486-3p) and negative control (NC). The cell apoptosis was detected according to the instructions of Annexin V-fluorescein isothiocyanate (FITC) kit (Sigma, St. Louis, MO, USA). Double staining with FITC-conjugated annexin V and propidium iodide (PI) was performed as follows. Forty-eight hours post transfection, the cells, including floating cells, were harvested, washed twice with 4 °C PBS, and resuspended in binding buffer (10 mM HEPES/NaOH, 140 mM NaCl, 2 mM KCl). Annexin V was added for 15 min in the dark. Cells were then washed, centrifuged, and resuspended in binding buffer. Before flow cytometric analysis, PI was added to each sample. Annexin V+ /PI− cells were early apoptotic cells. Finally, the cell apoptosis detection was performed by a FACS Canto II flow cytometer (Becton Dickinson, Franklin Lakes, NJ, USA).

### Western Blot Analysis

Total protein was extracted from cells in one well of a 6-well plate (~ 1 × 10^6^ cells) following 24 h treatment with 100 µM TMZ as aforementioned and the concentrations were measured using a spectrophotometer (Bio-Rad Laboratories, Inc., Hercules, CA, USA) with a bicinchoninic acid protein assay kit (Beyotime Institute of Biotechnology, Shanghai, China). Protein at a concentration of > 5 µg/µl (50 µg in total) was separated by 10% or 12% SDS‑PAGE and was transferred onto polyvinylidene difluoride membranes (EMD Millipore, Billerica, MA, USA), followed by blocking with skimmed milk dissolved in TBS and 0.05% Tween-20 (TBST) for 1 h at room temperature. The membranes were incubated with primary antibodies including MGMT (sc-271154, Santa Cruz, CA, USA), caspase-3 (9661S, Cell Signaling Technology, USA), γ-H2AX (2577S, Cell Signaling Technology, USA), and H2AX (2572, Cell Signaling Technology, USA) at 4 °C overnight, washed three times with TBST and were incubated in horseradish peroxidase-conjugated secondary antibodies at 1:5000 dilution (sc-2004; Santa Cruz Biotech, Santa Cruz, CA, USA) for 1 h at room temperature after washing with TBST three times. Following washing, the protein bands were detected with ECL substrates or DAB Detection System (OriGene Technologies, Inc., Beijing, China). β-actin (sc-47778, Santa Cruz, USA) was used as a loading control, and all experiments were repeated three times. Quantitative analysis was performed using the Quantity One Software (version 4.6.2; Bio-Rad Laboratories, Inc.).

### Nude Mouse Tumorigenicity Assay

Tumor-bearing nude mice (weight: 18–20 g; Vital River, Beijing, China) were divided into three groups (six mice per group). U251-TR cells (2 × 10^6^) stably expressing miR-486-3p, miR-486-3p-inhibitor, and miR-NC were collected and injected subcutaneously into NOD/SCID mice. All mice were housed under standard specific pathogen-free conditions with a 12 h light/12 h dark cycle. All mice had free access to food and water. After 2 weeks of tumor growth, all mice were given TMZ + Allicin (60 mg/kg TMZ + 50 mg/kg Allicin, oral gavage). The mice were treated daily for 3 weeks. Mice were monitored visually and sacrificed when neurological signs appeared. Kaplan–Meier survival curves were calculated using GraphPad Prism 7.0 software. This animal experiment was approved by the Institutional Review Board of The Second Affiliated Hospital of Zhejiang Chinese Medical University.

### Statistical Analysis

Quantitative data are presented as means ± SEM. The statistical significance levels were set at *P < 0.05 and **P < 0.01. One-way ANOVA and Student’s *t* tests were used to perform comparisons. Biostatistical analysis was performed using Office SPSS software (SPSS version 22.0) and GraphPad software (GraphPad Prism 5). All statistical tests were two-sided.

## Results

### Allicin Enhances the Sensitivity of U251-TR Cells to TMZ and Promotes Apoptosis

**Fig. 1:**
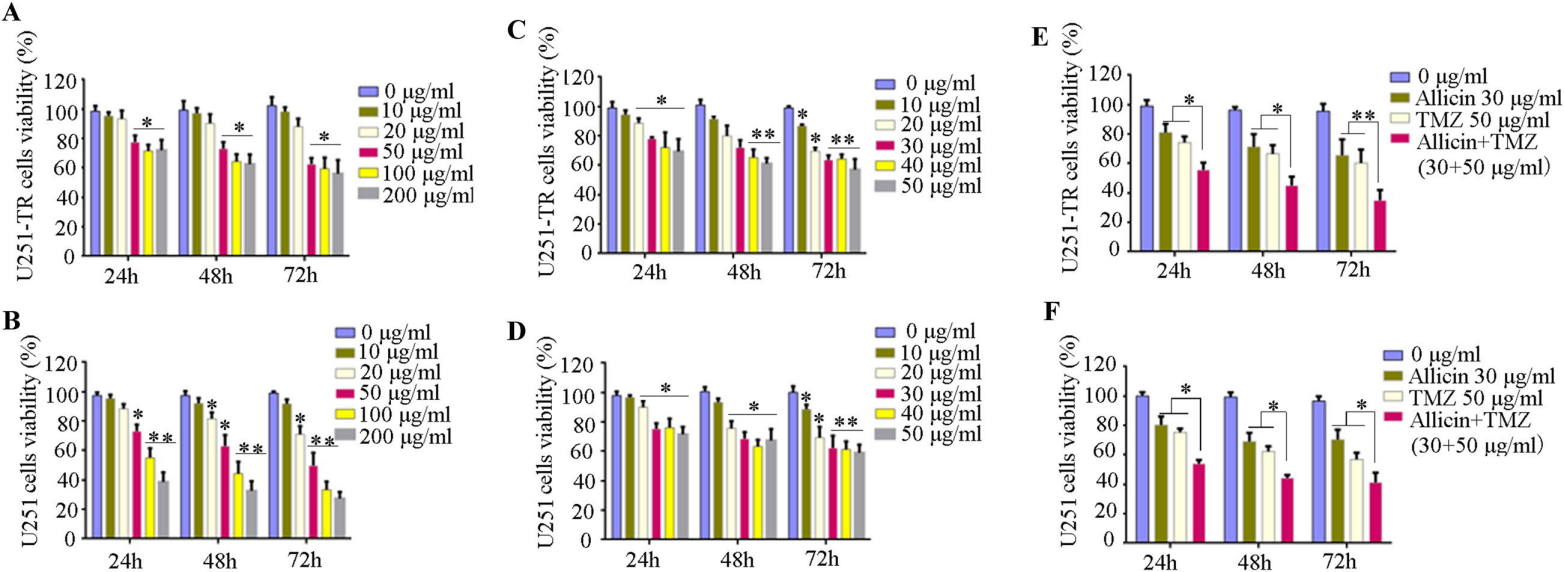
Allicin enhances the sensitivity of U251-TR cells to TMZ. **a**, **b** Effects of TMZ on U251-TR and parental U251 cells viability. U251-TR and parental U251 cells were treated with different concentrations (0, 10, 20, 50, 100, and 200 μg/ml) of TMZ for 24, 48, or 72 h. *P < 0.05, **P < 0.01 versus control group (0 μg/ml). **c**, **d** Effects of Allicin on U251-TR and parental U251 cells viability. U251-TR and parental U251 cells were treated with different concentrations (0, 10, 20, 30, 40, and 50 μg/ml) of Allicin for 24, 48, or 72 h. *P < 0.05, **P < 0.01 versus control group (0 μg/ml). **e**,** f** U251-TR and parental U251 cells were treated with 30 μg/ml Allicin: 50 μg/ml TMZ, 30 μg/ml Allicin + 50 μg/ml TMZ for 24, 48, or 72 h. *P < 0.05, **P < 0.01. Data are mean ± SEM of cell viability from three independent experiments with triplicates in each experiment

We firstly established TMZ-resistant GBM cell lines with increased concentrations of TMZ for different time periods (TMZ-resistant U251 cell lines: U251-TR). U251-TR and Parental U251 cells were treated with different concentrations (0, 10, 20, 50, 100, and 200 μg/ml) of TMZ for 24, 48, or 72 h. Results from CCK-8 assays showed that U251-TR cell viability was not affected when TMZ concentrations were < 50 μg/ml within 24 h. Whereas when the TMZ was increased to 200 μg/ml or extended to 72 h, there was no significant decrease in cell viability compared to the 50 μg/ml (Fig. 1a, b). However, parental U251 cell viability significantly decreased in a dose-and time-dependent manner. These results indicated that TMZ-resistant GBM cell line (U251-TR) was successfully established. In addition, U251-TR and Parental U251 cells were treated with different concentrations (0, 10, 20, 30, 40, and 50 μg/ml) of Allicin for 24, 48, or 72 h. Results showed that Allicin did not affect cell viability at concentrations less than 20 μg/ml within 24 h, whereas higher concentrations (> 30 μg/ml) of Allicin inhibited both U251-TR and parental U251 cell growth at 48 or 72 h (Fig. 1c, d). So 30 μg/ml Allicin, 50 μg/ml TMZ, or combination (30 μg/ml Allicin + 50 μg/ml TMZ) were used in the following study.

To determine whether Allicin can enhance the sensitivity of glioblastoma to TMZ. U251-TR and parental U251 cells were treatment with 50 μg/ml TMZ and/or 30 μg/ml Allicin for 24, 48, or 72 h, The results showed that 30 μg/ml Allicin + 50 μg/ml TMZ can significantly decrease parental U251 and U251-TR cells viability in time-dependent manner (Fig. 1e, f). Furthermore, primary glioblastoma cells were also used to confirm this effect, the results showed that 30 μg/ml Allicin + 50 μg/ml TMZ also can significantly decrease primary glioblastoma cells viability (Supplementary Fig. 1).

**Fig. 2:**
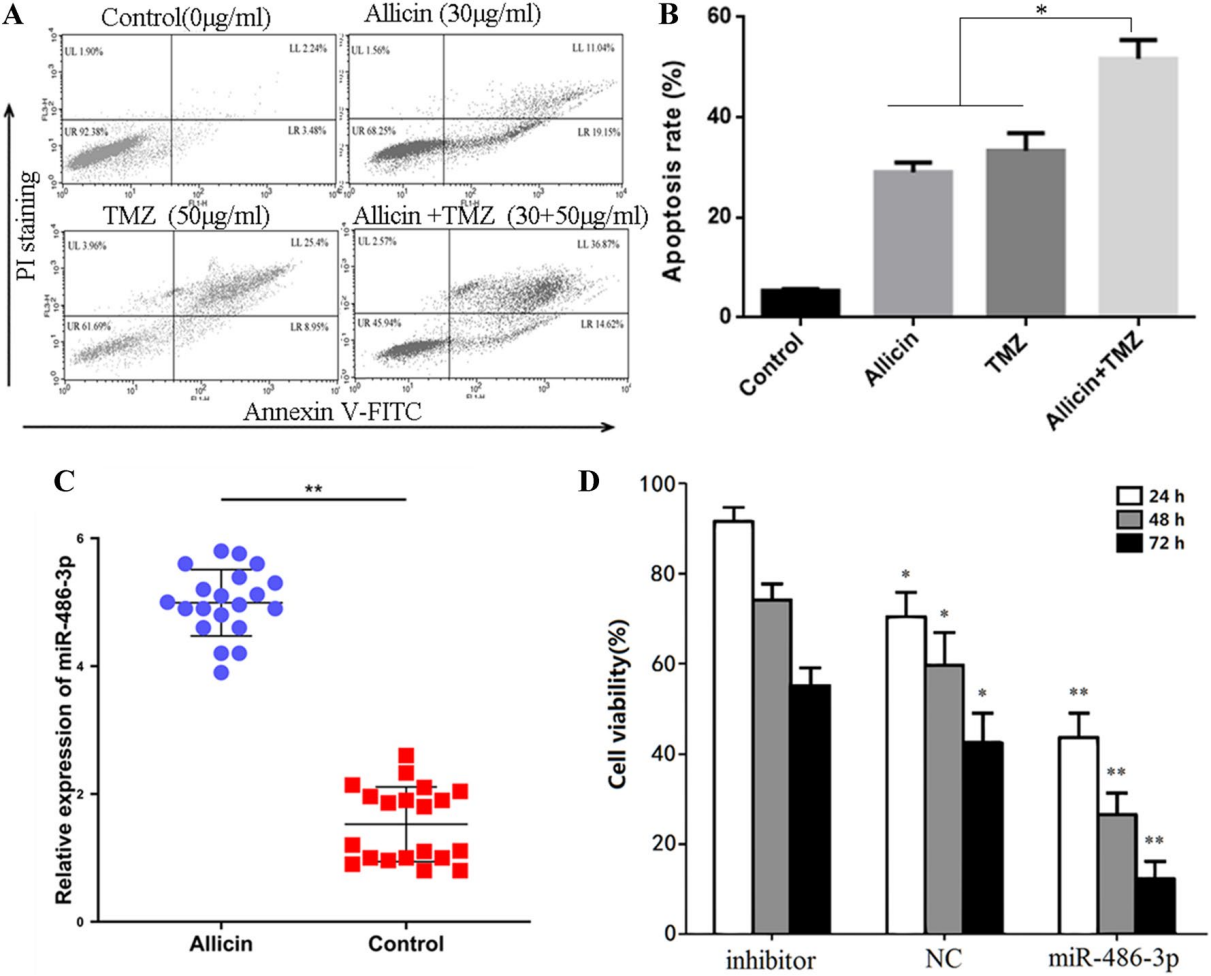
Allicin enhances the sensitivity of U251-TR cells to TMZ and promotes apoptosis and the expression of miR-486-3p. **a** Flow cytometry analysis showed that TMZ + Allicin group could significantly enhance apoptosis in cells after 48 h. **b** PCR analysis of miR-486-3p expression levels in Allicin group and control group. **c** Cell viability analyzed after treatment with Allicin + TMZ in control, miR-486-3p group, miR-486-3p-inhibitor group. Data are mean ± SEM from three independent experiments with triplicates in each experiment. *P < 0.05, **P < 0.01

In addition, Annexin V-FITC/PI staining assay was used to measure the apoptosis. The results in Fig. 2a show that compared with control, the percentage of apoptotic cells in combination (30 μg/ml Allicin + 50 μg/ml TMZ) group significantly increased from 5.78% of the control, 30.19% of 30 μg/ml Allicin, or 34.35% of 50 μg/ml TMZ to 51.49% (Fig. 2b). Taken together, these results indicated that Allicin can enhance the sensitivity of glioblastoma to TMZ.

### Allicin Enhances the Sensitivity of U251-TR Cells to TMZ by Promoting the Expression of miR-486-3p

Previous study showed that high expression of miR-486-3p may enhance the sensitivity of neuronal tumors to chemotherapeutic drugs. So we explored the levels of miR-486-3p in Allicin group and control group using qRT-PCR. The results showed that miR-486-3p levels were higher in Allicin group than control group (Fig. 2c). In order to verify whether Allicin improves the sensitivity of U251-TR to TMZ by regulating miR-486-3p, U251-TR cells were transfected with lentivirus vectors carrying miR-486-3p or inhibitor. An empty virus was used as positive transfection control (NC) group, and all groups were treated by 30 μg/ml Allicin + 50 μg/ml TMZ. As the results demonstrated, miR-486-3p group had lowest cell viability, but miR-486-3p-inhibitor group had highest cell viability (Fig. 2d). This indicated that Allicin could promote miR-486-3p overexpression, then enhances TMZ sensitivity in glioblastoma.

### MiR-486-3p Directly Targets MGMT

**Fig. 3:**
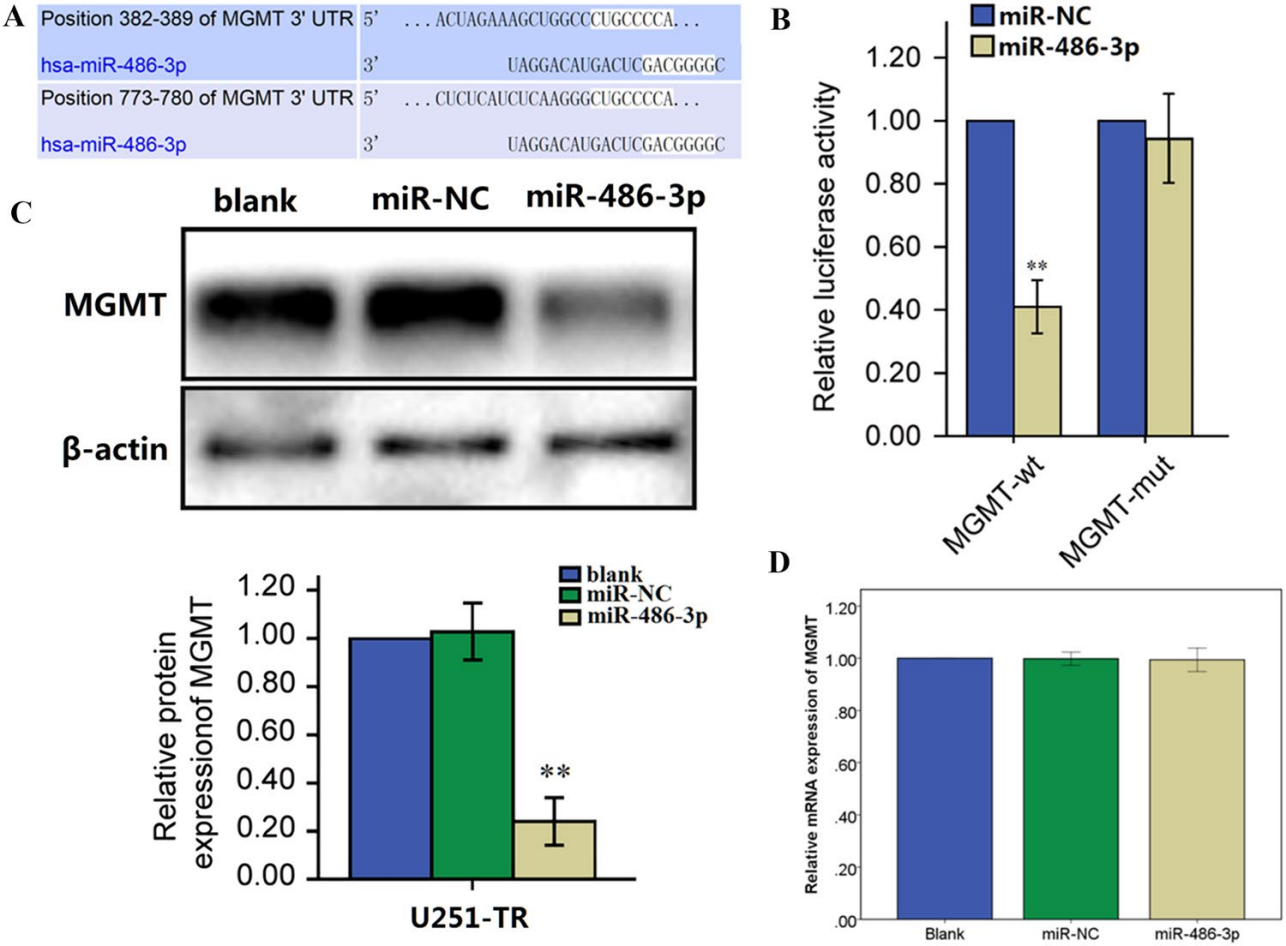
miR-486-3p directly targets MGMT. **a** The sequence of miR-486-3p binding sites within MGMT. The reporter constructs of the MGMT 3′-UTR sequences. **b** Luciferase reporter assay was performed to detect the relative luciferase activities of WT and Mut MGMT reporters. **c** The protein levels of MGMT in U251-TR cells overexpressing miR-486-3p or miR-NC. **d** The mRNA levels of MGMT in U251-TR cells transfected with miR-486-3p or miR-NC. Each data point represents the mean ± SEM from three independent experiments. *P < 0.05, **P < 0.01

To better understand the underlying function and mechanism of miR-486-3p in glioblastoma progression, several potential target genes of miR-486-3p were detected using the bioinformatic algorithm TargetScan (https://www.targetscan.org/). Among the candidates, we detected a predicted binding site for miR-486-3p in the 3′-UTR of MGMT (Fig. 3a). To verify whether MGMT is a novel direct target of miR-486-3p in glioblastoma, a luciferase assay was performed in pGL3-control luciferase reporter plasmids containing the putative wild-type (WT) and mutant (Mut) MGMT 3′-UTR binding site. U251-TR cells were treated with reporter vector (MGMT-WT or MGMT-Mut) and miR-NC (negative control) or miR-486-3p. Cells co-transfected with miR-486-3p and MGMT-WT reporter showed a noteworthy reduction of luciferase activities (Fig. 3b).

To further verify whether miR-486-3p regulates MGMT expression through mRNA degradation or translational repression, we established U251-TR cells stably expressing miR-486-3p or miR-NC. And the protein levels of MGMT were downregulated in cells stably expressing miR-486-3p (Fig. 3c). However, miR-486-3p overexpression did not affect the mRNA levels of MGMT (Fig. 3d). These results indicated that miR-486-3p directly targeted MGMT by binding to the 3′-UTR of MGMT and inhibited the translation of the MGMT mRNA into the MGMT protein in glioblastoma cells. These findings suggest a possible relationship between miR-486-3p expression and TMZ resistance.

### MiR-486-3p Overexpression Increases Chemosensitivity to Temozolomide

**Fig. 4:**
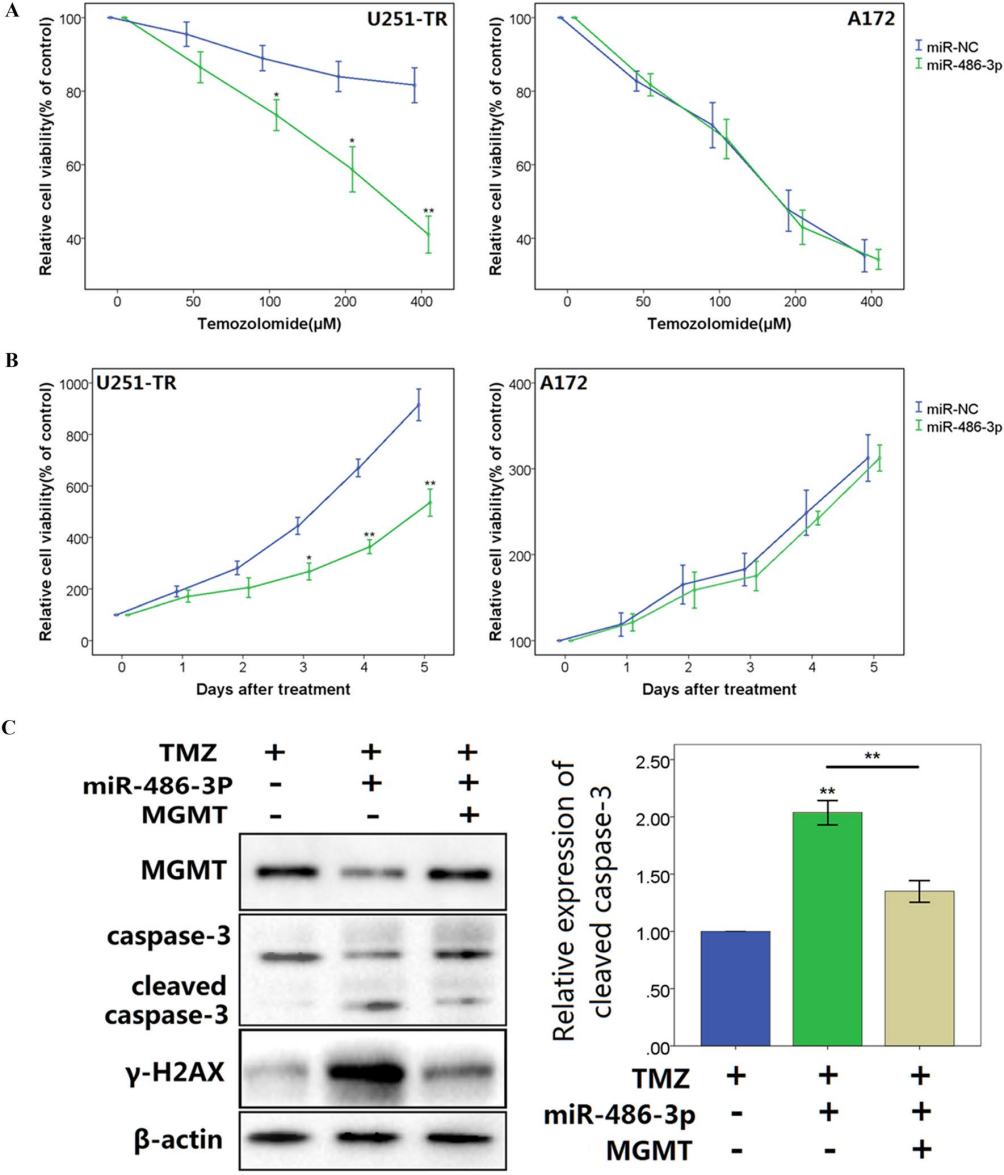
miR-486-3p overexpression increases chemosensitivity to temozolomide. **a** Cell proliferation was evaluated in U251-TR and A172 cells stably expressing miR-NC or miR-486-3p with the TMZ treatments at different concentration. CCK8 assay was performed 48 h after treatment. **b** Cell proliferation in 200 μM TMZ treatments was tested every 24 h in U251-TR and A172 cells overexpressing miR-486-3p and miR-NC. **c** Western blot analysis of MGMT, cleaved caspase-3, and γ-H2AX expression in U251-TR cells co-transfected with miR-486-3p or miR-NC, and cultured in 200 μM TMZ. The data represent results from one of three independent experiments. *P < 0.05, **P < 0.01

A172 cells are TMZ-sensitive cells lacking MGMT expression. We treated A172 cells and U251-TR cells stably expressing miR-486-3p or miR-NC with different concentrations of TMZ. As shown in Fig. 4a, CCK8 assay detected a significant decrease in cell viability following TMZ exposure in inverse correlation to TMZ concentration. Overexpressing miR-486-3p only increased chemosensitivity to TMZ in U1251-TR cells, but not in A172 cells. Next, we measured the viability of glioblastoma cells treated with TMZ (200 μg/ml) using CCK8 assay at different time points. The results showed that miR-486-3p overexpression significantly inhibited cell survival of U251-TR cells in the presence of TMZ (Fig. 4b). However, overexpression of miR-486-3p did not affect cell survival of A172 cells, indicating that miR-486-3p sensitized glioblastoma cells to TMZ might through MGMT. In addition, after TMZ treatment, glioblastoma cells expressing miR-486-3p had a significant decrease in the expression of MGMT and increase in the levels of phosphorylated histone H2AX (γ-H2AX, Markers of DNA double strand breakage) and cleaved caspase-3 compared with vector control cells. However, ectopic expression of MGMT significantly reversed the enhancement effect of miR-486-3p on γ-H2AX and cleaved caspase-3 in the presence of TMZ (Fig. 4c).

### MiR-486-3p Overexpression Enhances Temozolomide-Induced Growth Inhibition In Vivo

**Fig. 5:**
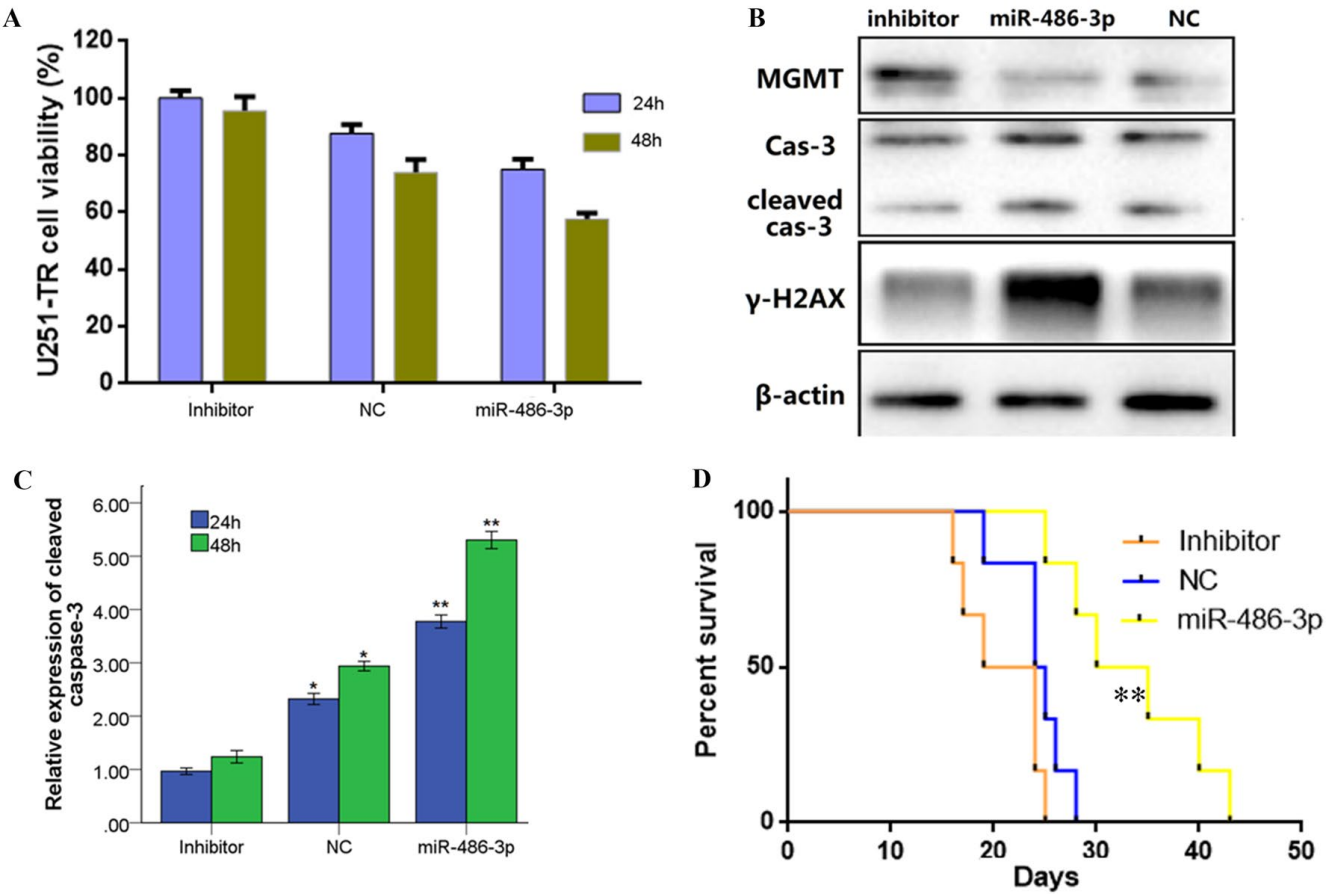
Allicin promotes high expression of miR-486-3p that enhances TMZ sensitivity in tumor-bearing mice. **a** Overexpression miR-486-3p reduced the U251-RT cells viability. Data presented as mean ± SEM of cell viability from three independent experiments with triplicates in each experiment. **b** Western blot analysis confirmed that after 48 h treatment, miR-486-3p-overexpression group and miR-NC group had a significant decrease in MGMT expression.**c** Expression analysis of cleaved caspase-3 showed that miR-486-3p overexpression group and miR-NC group promote apoptosis significantly. The data represent results from one of three independent experiments. **d** Survival curve of miR-486-3p overexpression, miR-486-3p inhibitor, and miR-NC control groups. *P < 0.05, **P < 0.01 versus miR-486-3p inhibitor group

In order to further confirm that MiR-486-3p overexpression enhances TMZ-induced growth inhibition, U251-TR cells were stably transfected with miR-486-3p, miR-486-3p-inhibitor, and miR-NC, respectively. CCK8 assay showed that after treatment with 30 μg/ml Allicin + 50 μg/ml TMZ for 24 or 48 h, the viability of U251-TR cells transfected with miR-486-3p or miR-486-3p-inhibitor was significantly decreased compared with miR-486-3p-inhibitor group (Fig. 5a). Western blot showed that compared with miR-486-3p-inhibitor group, the expression of MGMT was decreased, whereas cleaved caspase-3 (cleaved cas-3) and γ-H2AX expression were increased (Fig. 5b). In addition, after treatment with 30 μg/ml Allicin + 50 μg/ml TMZ for 24 or 48 h, the activities of caspase-3 significantly increased (Fig. 5c). We also used tumor-bearing mice to investigate whether Allicin can reverse TMZ resistance in vivo. The survival curves showed that in miR-486-3p-overexpression group and miR-NC groups, the survival time was significantly increased compared with miR-486-3p-inhibitor group (Fig. 5d). Together, these data suggest that Allicin could upregulate miR-486-3p and then lead U251-TR cells sensitize to TMZ in vivo.

## Discussion

Oral alkylating agent TMZ is a chemotherapeutic drug with remarkable effects on various solid tumors. At present, TMZ is listed as the first-line chemotherapeutic drug for glioblastoma. TMZ simultaneous radiotherapy combined with adjuvant chemotherapy is the standard treatment for glioblastoma. However, after years of clinical observation, its effect is still limited. The reasons may be as follows: First, the presence of the blood–brain barrier affects the distribution and absorption of the drug in the brain. Second, it may be due to tumor resistance genes, which made tumor cells lose sensitive to TMZ and drug resistance, resulting in tumor progression or recurrence (Arora and Somasundaram 2019; Shea et al. 2016). Therefore, it is meaningful to research the mechanism of TMZ resistance in glioblastoma and try to reverse it.

Previous study reported that Allicin suppresses the growth of certain cancers in vitro and in vivo (Dvořáková et al. 2015). Consistent with this report, our study showed that Allicin can increase the sensitivity of U251-TR to TMZ and promote glioblastoma cells apoptosis. Further study showed that the expression of miR-486-3p was significantly increased in Allicin treated group than control group. Based on bioinformatics analysis, it is suggested that high expression of miR-486-3p may enhance the sensitivity of chemotherapeutic drugs in glioblastoma (Plaisier et al. 2016). The results of our study justify this hypothesis and found for the first time that Allicin can promote glioblastoma cells apoptosis by promoting the high expression of miR-486-3p, reversing TMZ resistance. This finding complements previous research (Chu et al. 2012; Lawson and Wang 2005; Pan et al. 2018; Oommen et al. 2004) and partially clarifies the anti-tumor effect of Allicin. Nevertheless, its role in glioblastoma is poorly understood.

Many studies have demonstrated that the most popular mechanism of TMZ resistance is the expression of O6-methylguanine-DNA methyltransferase (MGMT) (Wick et al. 2014). MGMT protein is a kind of DNA repair enzyme, which can protect normal tissues from alkylating agent damage, reduce cancer, and also cause tumor tissue resistance to alkylating agent chemotherapy. There have been many studies on the effects of MGMT on TMZ resistance in glioblastoma treatment. Some researchers have found that cytotoxic drug withaferin A, which is involved in oxidative stress, can increase the sensitivity of glioblastoma to TMZ treatment by reducing MGMT expression and activating apoptosis induced by Akt/mTOR signaling pathway (Grogan et al. 2014). miR-200a-3p has also been found to interact with MGMT to increase susceptibility of glioblastoma to chemotherapy drugs (Berthois et al. 2014). Some studies found that liver-type glutaminase (LGA), which is transfected and expressed in human glioblastoma cell lines, causes a decrease in MGMT expression, and also increases sensitivity of glioblastoma to chemotherapy drugs (Szeliga et al. 2012). And the expression of MGMT is upregulated by a variety of mechanisms, such as the acetylation of histones H3 and H4 (Nakagawachi et al. 2003), different nuclear transcription factors (AP-1, HIF-1α, CEBP, Sp1, and NF-κB) (Bocangel et al. 2009), and the stabilization by binding of N-myc downstream regulated gene 1 protein (Weiler et al. 2014). The present study showed that miR-486-3p could directly bound to the 3′-UTR of MGMT, decreased the protein expression of MGMT through inhibiting the translation of the MGMT mRNA into the MGMT protein. Meanwhile, miR-486-3p overexpression rendered glioblastoma cells more sensitive to TMZ through targeting MGMT.

In summary, this study suggest that Allicin can increase the expression of miR-486-3p in glioblastoma, and miR-486-3p inhibits protein translation of MGMT, which reverses TMZ resistance and promotes glioblastoma apoptosis in TMZ environment. Our study further elucidate the biological function of Allicin in glioblastoma. This biological function indicates that, in the future, Allicin can be used as adjunctive drug for TMZ to improve patient prognosis, and miR-486-3p may be a potential target for glioblastoma treatment, improving the effectiveness of comprehensive treatment.

## Electronic supplementary material

Below is the link to the electronic supplementary material.

Supplementary file1 (TIF 2233 kb)
