# Supplementary figures and images for: Overexpression miR-486-3p Promoted by Allicin Enhances Temozolomide Sensitivity in Glioblastoma Via Targeting MGMT
Source: Neuromolecular Med. 2020 Feb 21;22(3):359–69. doi: 10.1007/s12017-020-08592-5 (PMC7417398; doi:10.1007/s12017-020-08592-5)

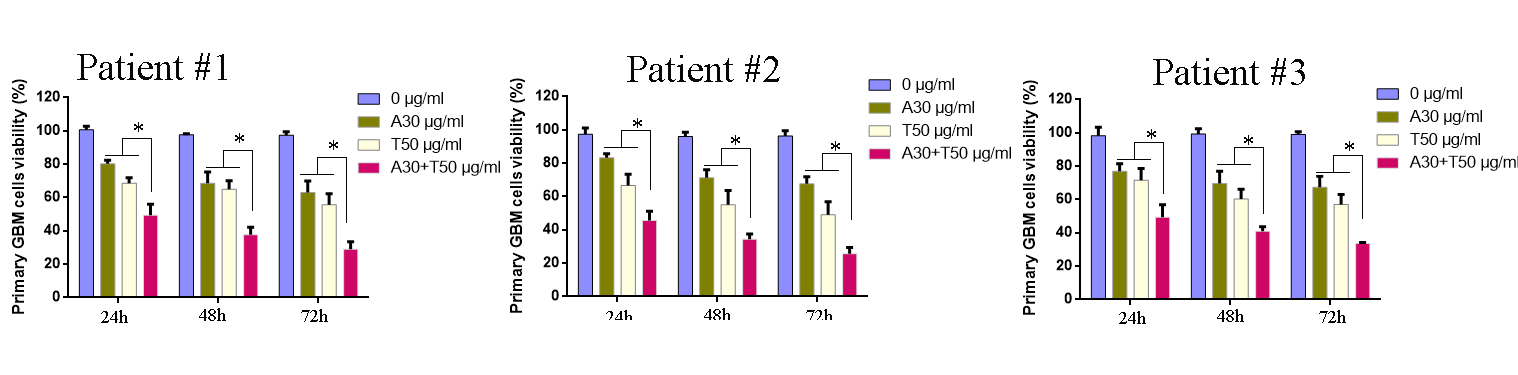

Supplement: Supplementary file 1 — Supplementary file1 (TIF 2233 kb) [file 12017_2020_8592_MOESM1_ESM.tif]
